# Intensive care admission of cancer patients: a comparative analysis

**DOI:** 10.1002/cam4.430

**Published:** 2015-04-18

**Authors:** Monique M E M Bos, Ilona W M Verburg, Ineke Dumaij, Jacqueline Stouthard, Johannes W R Nortier, Dick Richel, Eric PA van der Zwan, Nicolette F de Keizer, Evert de Jonge

**Affiliations:** 1 Department of Medicine, Reinier de Graaf HospitalDelft, The Netherlands; 2 Department of Medical Informatics, Academic Medical Center, University of AmsterdamAmsterdam, The Netherlands; 3 Department of Medical Oncology, Antoni van Leeuwenhoek Hospital, Dutch Cancer InstituteAmsterdam, The Netherlands; 4 Department of Medical Oncology, Leiden University Medical CenterLeiden, The Netherlands; 5 Department of Medical Oncology, Academic Medical Center, University of AmsterdamAmsterdam, The Netherlands; 6 Department of Intensive Care, Leiden University Medical CenterLeiden, The Netherlands

**Keywords:** Age differences, cancer, gender differences, intensive care, survival

## Abstract

The aim of this study was to obtain insight into which proportion of cancer patients is admitted to an Intensive Care Unit (ICU) and how their survival, demographic, and clinical characteristics relate to cancer patients not admitted to the ICU. Data from patients registered with cancer between 2006 and 2011 in four hospitals in the Netherlands were linked to the Dutch National Intensive Care Evaluation registry. About 36,860 patients with cancer were identified, of whom 2,374 (6.4%) were admitted to the ICU. Fifty-six percent of ICU admissions were after surgery, whereas 44% were for medical reasons. The risk for ICU admission was highest among cancer patients treated with surgery either alone or combined with chemotherapy and/or radiation therapy. Only 80 of 1,073 medical ICU admissions (3.3%) were for cancer-specific reasons. Although more women (54.0%) than men were registered with cancer, the proportion of male cancer patients admitted to an ICU was much higher (9.3 vs. 4.0%, *P* < 0.001). Five-year survival of cancer patients admitted to the ICU was substantial (41%) although median survival was much lower (1,104 days) than in patients not admitted to the ICU (median survival time not reached, *P* < 0.001). These results show that one out of 16 cancer patients was admitted to an ICU and that ICU support for this group should not be considered futile.

## Introduction

The prognosis of cancer patients has improved due to earlier diagnosis and better therapeutic options 1. As a consequence of more aggressive therapies, the need of cancer patients for intensive care unit (ICU) support during the course of their disease has increased 2. Recent data indicate that the outcome of cancer patients on the ICU has improved significantly 3,4. ICU admission is now considered appropriate for patients with a malignancy for specified indications such as postoperative care, complications caused by the malignancy and/or its treatment and crises unrelated to the tumor or its therapy 5,6.

Previous investigations reported on the incidence of cancer and its impact on the outcome in general ICUs 7–9. These studies showed that a cancer diagnosis on admission to a general ICU is relatively common, varying between 13.5% and 21.5%, and that the outcome of these patients is strongly dependent on the type of admission, with planned surgical and unplanned medical admission types bearing the relatively best and worst prognosis, respectively 7–9.

Although information about the influence of cancer on ICU outcome has become increasingly available during recent years 3,4,7–10, knowledge on the proportion and characteristics of cancer patients from a general population that is admitted to an ICU is highly limited. This information is of considerable interest, not only for insight in epidemiology and health care costs associated with different cancer diagnoses, but also for general and specialist physicians providing treatment and care to patients with a malignancy. The novelty of this study is that it sought to obtain insight into which proportion of cancer patients is admitted to an ICU during the course of their illness and to analyze differences between cancer patients who were and who were not admitted to the ICU with regard to demographics, cancer diagnosis, type of treatment, and outcome. We hypothesized that ICU admissions of cancer patients from a general population would predominantly involve patients undergoing major elective cancer surgery and patients suffering from cancer- and therapy-specific complications, but were not able to predict which percentage of patients with specific cancer and/or treatment types (surgery, chemotherapy, radiation) would be in need of ICU care most. We performed an observational study encompassing a 5-year period and involving 36,860 cancer patients, stratified according to cancer diagnosis and treatment type, in the Netherlands.

## Materials and Methods

### Patient selection

The primary population consisted of all adult patients with a cancer diagnosis registered between January 1, 2006 and January 1, 2011 in four hospitals in the Netherlands. Two academic hospitals (Academic Medical Center, Amsterdam and Leiden University Medical Center, Leiden) and two community teaching hospitals (Reinier de Graaf Hospital, Delft and Maasstad Hospital, Rotterdam) participated. Patients were selected from the hospital information systems based on encoded “Diagnosis Treatment Combinations” (DTC), a nationwide coding and reimbursement system providing information about the type of care, diagnosis and all treatment modalities specified by the attending physician 11,12. A DTC is opened upon first contact with a patient and selected based on specific guidelines 11,12. For each patient a DTC remains active as long as he or she receives treatment or is in follow-up for the specified diagnosis. One patient can have more than one DTC, for example in the presence of two different primary tumors. For the current analysis all patients with a DTC related to an oncological diagnosis were included, with the exception of patients with a primary malignancy of the central nervous system (since not all participating hospitals were primary care givers of this type of patients) or superficial skin cancer (since these tumors are not expected to lead to ICU admission or to influence survival). To identify patients who were admitted to an ICU during the study period, data were linked to the database of the Dutch National Intensive Care Evaluation (NICE) registry using a deterministic linkage algorithm 13,14 using name (encrypted surname and maiden name), date of birth, gender, and the comparison of ICU admission date and start- and end of an cancer period. NICE contains information on all admissions to the ICUs of 84 hospitals in the country (i.e., approximately 90% of all ICUs in the Netherlands) 15,16. As such, while the four hospitals from which the primary cancer population was retrieved participated in the NICE registry, ICU admissions of patients from this primary population were also taken into account if they occurred in other hospitals. Within NICE, several measures are taken to improve data quality, including mandatory training in data definitions and data collection and at least three-yearly site-visits with re-collection of data. Data quality in the NICE registry has been evaluated and confirmed published previously 15,17. In order to avoid patient identification, identifying variables are included in an encrypted form in NICE. For the current analysis, patient identifying information in the DTC database was encrypted based on the same encryption algorithm as used in NICE. ICU admissions were linked with patients from the primary population in case the DTC encoding cancer was either active or closed less than one year before ICU admission. The date of death was extracted from the hospital information systems. The participating hospitals obtain date of death information from the Dutch population registry. The date of death was updated until the complete follow-up period July 1, 2011. In case a patient is admitted at the ICU and died during the same hospitalization period the date of death is checked using information from the NICE registry.

### Ethics

The need for ethical committee approval was waived by the Dutch Central Committee on Research Involving Human Subjects, because the study was purely observational and because only deidentified patient data were used.

### Statistical analysis

Samples median test was used to test whether the median age differed for patients with and without ICU admission. Pearson chi-squared test was used to evaluate whether the distribution of the different categories of variables age, gender, phase of active cancer treatment, therapy combination, and type of cancer differed between cancer patients with and without ICU admission. Logistic regression using General Linear Models analyses was used to determine the contribution of the type of cancer on admission to the ICU during an active cancer diagnosis. The calculated odds ratios were adjusted for gender, phase of active cancer treatment, therapy combinations, age, and participating hospital.

For all cancer diagnoses combined a Kaplan–Meier curve was constructed for time to ICU admission. Cumulative percentages after 30, 365, and 730 days of ICU admission were obtained by calculating survival tables for type of cancer and gender. The log rank (Mantel-Cox) test was performed to test whether survival distributions differed between men and women. Time to ICU admission was defined as time from the first day of the first DTC until ICU admission. Patients were censored at one year after the last DTC was closed or at January 1, 2012, whatever came first.

To compare survival after the first diagnosis of cancer between patients with and without ICU admissions during an active cancer diagnosis Kaplan–Meier curves and Cox Proportional Hazard (Cox PH) regression were applied. The starting date of the first DTC was chosen as starting time, date of death was the endpoint. The calculated hazard ratio was adjusted for gender, phase of active cancer treatment, therapy combinations, types of cancer, and age by adding these covariates in the Cox PH regression model. Patients were censored at the end of follow-up period July 1, 2011.

Furthermore, to analyze survival after ICU admission for patients with one or more ICU admissions during an active cancer diagnosis, Kaplan–Meier curves were plotted for different types of cancer and for gender. Patients with a medical admission for cancer; a surgical admission for cancer and other admission diagnoses were compared in Kaplan–Meier curves and the log-rank (Mantel-Cox) test was performed to test whether survival distributions differed. For these analyses ICU admission date of the first ICU admission was chosen as starting time, date of death was the endpoint. Patients were censored at the end of follow-up period July 1, 2011. However, for ICU admissions that took place between July 1, 2011 and January 1, 2012 we used the hospital discharge date or death as endpoint, patients were censored when they survive their hospital stay.

Age was modeled using natural cubic regression splines 18, with four degrees of freedom in both Cox PH regression and logistic regression. The appropriate number of degrees of freedom was assessed by univariate analyses using analysis of variance. The proportional Hazard assumption was tested. For all analyses *P*-values below 0.05 were considered statistically significant.

All statistical analyses were performed using the statistical environment R version 2.14.1 (Amsterdam, The Netherlands) and PASW statistics 18.

## Results

### Patients and ICU admissions

**Figure 1:**
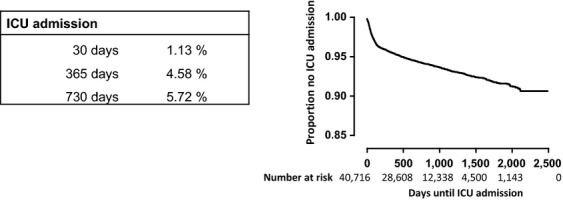
Kaplan–Meier curve for time until intensive care unit admission for all cancer diagnoses. The graph shows the time to ICU admission from opening of the diagnosis treatment combination for all 40,716 cancer diagnoses.

The primary population consisted of 36,860 patients with in total 40,716 cancer diagnoses, indicating that approximately 10% of patients had more than one cancer diagnosis (Table1). Between January 1, 2006 and January 1, 2011, 2,374 of these patients (6.4%) were admitted at least once to the ICU. Patients admitted to the ICU were older (median age 66 years) than patients not admitted to the ICU (median age 62 years, *P* < 0.001). The proportion of cancer patients admitted to the ICU was highest in the age group 60–75 years (8.1%) and lowest in the age group <45 years (2.8%). The risk of being admitted to an ICU was highest in patients treated with surgery (11.8%), surgery with radiation therapy (8.1%), surgery with chemotherapy (9.3%) and surgery combined with both chemotherapy and radiation therapy (10.5%). A more detailed analysis of the proportions of cancer patients admitted to an ICU by age and cancer therapy is given for specific cancer diagnoses in Tables S1 and S2. Figure1 shows the time from the first cancer diagnosis to ICU admission for all patients by Kaplan-Meier curve, revealing that most ICU admissions in cancer patients take place in the first year.

**Table 1:** Study population demographics

	All	ICU admission	No ICU admission	*P*^2^
	(% ICU admission)	(% within this group)	(% within this group)	
All patients	36,860 (6.4)	2,374	34,486	<0.001
Total number of cancer diagnosis	40,716 (6.0)	2,458	38,258	<0.001
Age (years), median (interquartile range)	63 (52–72)	66 (57–73)	62 (52–72)	<0.001
<45 years	5,186 (2.8)	143 (6.0)	5,043 (14.6)	<0.001
45–65 years	11,063 (5.8)	646 (27.2)	10,417 (30.2)	<0.001
>60–75 years	14,323 (8.1)	1,158 (48.8)	13,165 (38.2)	<0.001
>75 years	6288 (6.8)	427 (18.0)	5861 (17.0)	0.214
Gender				
Male	16,967 (9.3)	1,581 (66.6)	15,386 (44.6)	<0.001
Female	19,893 (4.0)	793 (33.4)	19,100 (55.4)	<0.001
Treatment (combinations^1^)				
Surgery	7,661 (11.8)	904 (36.8)	6,757 (17.7)	<0.001
Surgery and chemotherapy	2,044 (9.3)	190 (7.7)	1,854 (4.8)	<0.001
Surgery and radiation therapy	1,289 (8.1)	104 (4.2)	1,185 (3.1)	0.002
Surgery, chemotherapy, and radiation therapy	914 (10.5)	96 (3.9)	818 (2.1)	<0.001
Chemotherapy	3,426 (6.2)	213 (8.7)	3,213 (8.4)	0.137
Chemotherapy and radiation therapy	1,238 (7.0)	87 (3.5)	1,151 (3.0)	0.309
Radiation therapy	7,502 (2.3)	169 (6.9)	7,333 (19.2)	<0.001
Palliative care	911 (4.2)	38 (1.5)	873 (2.3)	0.017
Other/none	15,731 (4.2)	657 (26.7)	15,074 (39.4)	<0.001

1 Combinations of treatments are indicated independent of the order in which these were provided.

2 ICU admission versus no ICU admission.

### Different types of malignancy

The most frequent types of malignancy in the primary population were breast cancer (20.2% of all cancer diagnoses), hematological malignancy (10.5%), lung cancer (8.7%), colorectal carcinoma (8.3%), and prostate cancer (7.2%) (Table2). Esophageal cancer most commonly lead to ICU admission (27.3% of patients with this diagnosis were admitted within 730 days, Table2); In Table S3, the odds ratios for ICU admission are given for specific cancer diagnoses after adjustment for gender, cancer treatment, and age. In accordance, esophageal cancer was after adjustment still associated with the highest odds ratio (3.27) compared with lung cancer. Patients with other types of gastrointestinal cancer, including colorectal (10.4%) and pancreatic and biliary cancer (9.4%) also were relatively frequently admitted to the ICU (Table2).

**Table 2:** Cumulative intensive care unit admission stratified according to cancer diagnosis and gender

	Number			30 days ICU admission (%)^2^				365 days ICU admission (%)^2^				730 days ICU admission (%)^2^			
	*N* (%)^1^	Male	Female	All	Male	Female	*P*	All	Male	Female	*P*	All	Male	Female	*P*
Nongender-specific malignancy															
Lung cancer	3,546 (8.7)	2,125	1,421	1.6	2.0	1.1	0.474	5.3	6.2	4.0	0.061	6.3	7.5	4.4	0.003
Head and neck cancer	1,876 (4.6)	1,017	859	0.9	0.9	0.9	0.676	3.7	4.7	2.6	0.131	5.2	6.6	3.5	0.001
Colorectal cancer	3,389 (8.3)	1817	1,572	2.3	2.3	2.3	0.660	8.4	9.9	6.7	0.263	10.4	11.9	8.7	0.003
Pancreatic and biliary cancer	2,759 (6.8)	1,418	1,341	2.8	3.2	2.3	0.032	8.2	9.9	6.3	0.066	9.4	11.1	7.6	0.005
Esophageal cancer	1,661 (4.1)	1,274	414	2.8	2.3	4.1	0.765	25.5	26.7	22.0	0.214	27.3	28.7	23.1	0.102
Other types of GI-cancer	1,323 (3.2)	803	520	2.5	2.6	2.3	0.377	10.4	11.7	8.5	0.382	11.8	13.7	9.0	0.011
Urinary tract cancer	2,367 (5.8)	1,612	755	0.9	1.0	0.8	0.452	4.6	5.1	3.4	0.547	5.5	6.2	4.1	0.011
Melanoma	491 (1.2)	225	266	0.2	0.4	0.0	–3	0.8	1.8	0.0	0.050	1.5	2.5	0.7	0.059
Sarcomas	2,245 (5.5)	1,149	1,096	0.4	0.6	0.2	0.170	1.9	2.3	1.4	0.017	2.8	3.6	1.9	0.019
Hematological malignancy	4,275 (10.5)	2,370	1,905	1.6	1.9	1.3	0.675	4.7	5.8	3.3	0.332	6.2	7.7	4.4	<0.001
Other types of cancer	2,162 (5.3)	1,048	1,114	1.4	1.8	1.1	0.293	3.3	4.0	2.6	0.193	4.2	5.4	3.0	0.201
Gender-specific malignancy															
Breast cancer	8,241 (20.2)			0.1				0.5				1.2			
Ovarian and endometrial cancer	1,598 (3.9)			0.8				1.4				1.5			
Cervical cancer	1,015 (2.5)			0.0				0.3				0.9			
Other types of gyn. cancer	446 (1.1)			0.0				0.4				0.8			
Prostate cancer	2,927 (7.2)			0.1				1.3				3.0			
Testicular cancer	395 (1.0)			0.0				1.0				1.6			

1 Percentage of all cancer diagnoses.

2 Percentage of type of cancer in this cohort of 40,716 cancer diagnoses.

3 No comparison analysis is performed because the factor variable has only one value for every stratum.

### Indications for ICU admissions

According to the APACHE IV ICU admission diagnoses, 56.2% of ICU admissions were surgical. Only 26.9% of ICU admissions were directly linked with cancer (Table3). About 23.6% of all ICU admissions of patients were after surgery for cancer, whereas 32.6% of admissions were associated with surgery not directly related to the cancer diagnosis. Almost half of admissions to the ICU was for nonsurgical reasons. Only 3.3% of ICU admissions were medical and directly linked with cancer; the majority of medical admissions was because of infection and sepsis (18.5% of all ICU admissions of cancer patients).

**Table 3:** Reason for admission in the ICU

Type of ICU admission based on APACHE IV	Number of patients (%)
Surgery for cancer	579 (23.6)
Other types of surgery	
Gastro-intestinal surgery	322 (13.1)
Respiratory tract surgery (other than cancer)	163 (6.6)
Cardiovascular surgery	196 (8.0)
Other	121 (4.9)
Medical cancer	80 (3.3)
Other types of nonsurgical admissions	
Cardiac	173 (7.0)
Respiratory	143 (5.8)
Infection/sepsis	455 (18.5)
Thrombosis/hemorrhage	91 (3.7)
Neurological	44 (1.8)
Other nonsurgical causes for ICU admission	87 (3.5)
Missing	4 (0.2)

### Gender differences

The primary population of cancer patients comprised more women (54.0%) than men (Table1). In contrast, in the ICU group the proportion of men was much higher (66.6%, vs. 44.6% men in the non-ICU group, *P* < 0.001). This gender difference with more men than women admitted to the ICU was present in all cancer diagnosis-specific subgroups, although differences for esophageal cancer and melanoma and ‘other types of cancer’ did not reach statistical significance (Table2).

### Mortality

**Figure 2:**
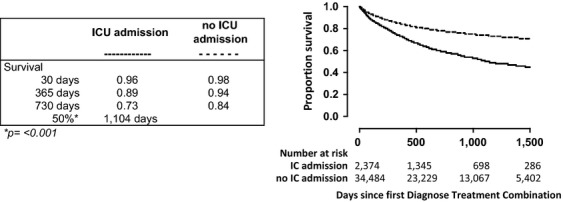
Survival of cancer patients who were or were not admitted to the ICU. Survival of cancer patients who were (solid line) or who were not (dotted line) admitted to the ICU. This analysis encompassed 36,860 adult patients registered with a cancer diagnosis between January 1, 2006 and January 1, 2011 in four hospitals in the Netherlands. Of these patients, 2374 were admitted to the ICU during the study period. The graph shows Kaplan–Meier curves starting at the date of cancer diagnosis registration; p value indicates the difference between groups.

**Figure 3:**
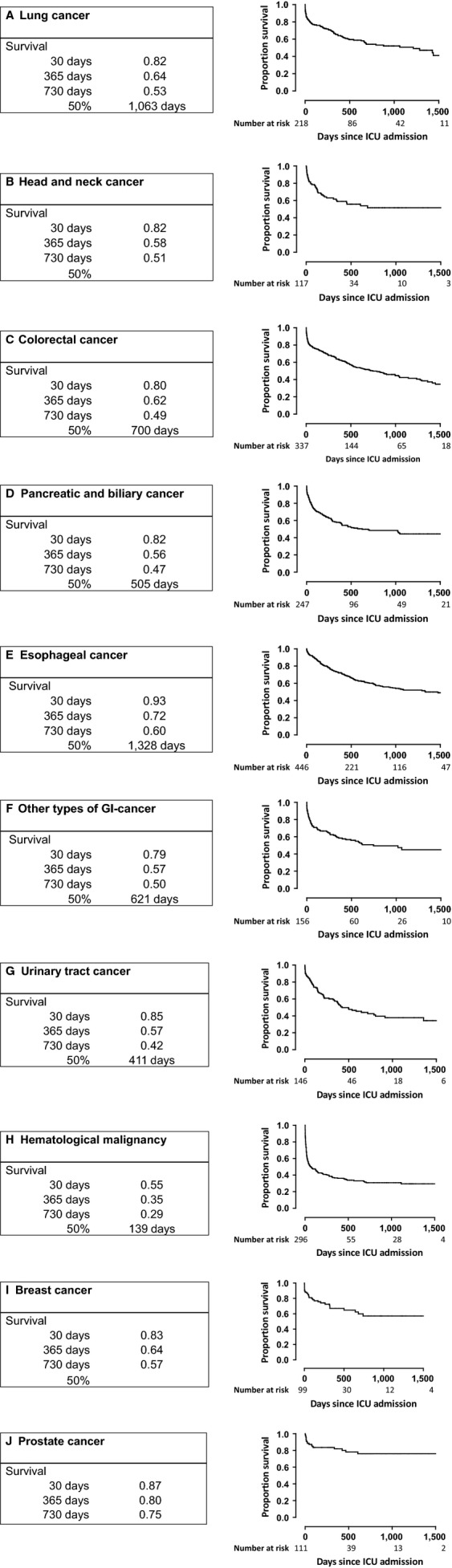
Kaplan–Meijer survival curves of cancer patients admitted to the ICU according to type of cancer. (A) Lung cancer, (B) Head and neck cancer, (C) Colorectal cancer, (D) Pancreatic and biliary cancer, (E) Esophageal cancer, (F) Other types of gastrointestinal cancer, (G) Urinary tract cancer, (H) Hematological malignancy, (I) Breast cancer, (J) Prostate cancer.

**Figure 4:**
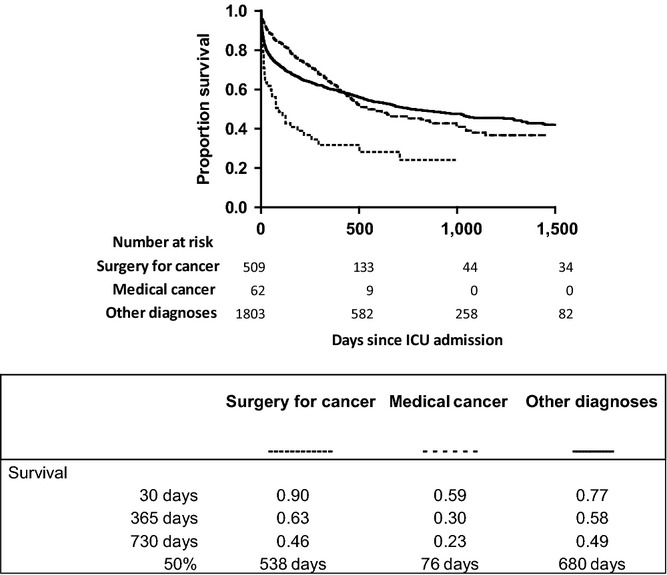
Survival of patients admitted to an ICU coded as medical admission for cancer, surgical admission for cancer; or other admission diagnosis.

Figure2 shows 5-year mortality stratified according to ICU admission status. Clearly, patients who were admitted to the ICU had a strongly reduced survival (median survival time 1104 days) when compared with patients who were not admitted to the ICU (median survival time not reached during the observation period; *P* < 0.001). Amongst cancer patients admitted to the ICU median survival times were 789 days for men and 574 days for women (not significant). Figure3A–J shows survival of patients admitted to the ICU stratified according to cancer diagnosis. Median survival times were relatively long for prostate cancer and breast cancer (not reached within 5 years) and esophageal cancer (1,328 days), and relatively short for hematological malignancies (139 days). Figure4 shows survival of patients admitted to an ICU coded as medical admission for cancer; surgical admission for cancer; or other admission diagnosis. The survival curves did not significantly differ.

## Discussion

To the best of our knowledge, our study is the first to report on the proportion of cancer patients from the general population that is admitted to an ICU during the course of their illness, stratified according to cancer diagnosis and type of treatment. Of 36,860 patients registered in four hospitals with at least one cancer diagnosis 6.4% was admitted to the ICU. Surgery was the most common treatment associated with ICU admission: of all cancers treated with surgery solely or in combination with other modalities 10.9% required ICU care. Among different cancer diagnoses, patients with esophageal cancer entered the ICU most frequently (27.3%).

Previous studies have documented that most ICU admissions of cancer patients involve postoperative care after elective surgery 14,19,20. Accordingly, our data show that the majority of cancer patients entered the ICU during the course of their disease because of surgical treatment. Of interest, however, of all cancer patients who were admitted to the ICU for surgical reasons, only 41.9% of cases involved surgical treatment directly related to cancer. Hence, more than half of cases were related to surgical interventions not directly linked with the primary cancer diagnosis, most notably gastrointestinal and cardiovascular surgery. In accordance with previous investigations 9,21–23, infection and sepsis were the most common indications for admission in medical cancer patients.

Although in this population of cancer patients women were more prevalent than men, the proportion of men that entered the ICU was more than twice as large when compared with women. While for the overall population this gender difference can be partly explained by the high prevalence of breast cancer (which very rarely results in ICU admission), men more often entered the ICU across all cancer diagnoses. Previous studies also indicated that men are more likely to receive ICU care 24–27. A large population-based study conducted in Canada demonstrated that older women with critical illness were less likely than critically ill men to be admitted to an ICU 26. Similarly, a prospective study involving 25,998 adult patients admitted to 31 ICUs in Austria documented gender-related differences in ICU care, with male patients—despite presenting with a lower severity of illness—more likely than female patients to receive a high level of care, as defined by the number of invasive procedures 25. Although in that investigation women had a higher observed mortality rate than men, there was no difference in outcome after adjustment for the severity of illness 25. Overall ICU mortality did not differ between sexes in another study 28. Our current study extends these data by showing that long-term survival of cancer patients admitted to the ICU does not differ between men and women. At present it is unclear what drives the apparent gender difference with regard to ICU admission of cancer patients, although some studies have suggested that males have more comorbid conditions at the point of cancer diagnosis, which may drive more frequent ICU admission 29–32.

More than 60% of cancer patients have an expected survival beyond 5 years 1,33, illustrating the need to study long-term outcome in patients with a malignancy. Previous studies of long-term survival of ICU patients have suggested an increase in mortality during several years after hospital discharge when compared with an age- and gender-matched population 34,35. Among different diagnoses, cancer patients had the greatest relative risk of mortality (more than threefold) during 5 years after hospital discharge following ICU admission in a cohort of 12,180 ICU patients from 25 hospitals in Finland 35. In accordance, the presence of a new malignancy was associated with a high risk of death within the first year after discharge (hazard ration 4.60) in a single-center study conducted in Australia comprising almost 20,000 ICU patients who survived to discharge 36. A more recent study conducted in the Netherlands analyzed mortality up to 3 years after hospital discharge of patients who had received ICU care. Whereas 1- and 3-year mortalities after hospital discharge were 12.5% and 27.5% respectively for the total ICU population, in cancer patients mortality was almost twice as high at both time points 14; these data are in line with our current long-term survival data of cancer patients after ICU admission. We also analyzed mortality up to 5 years after ICU admission stratified according to cancer diagnosis. Of note, 5-year survival rates of this subgroup of patients admitted to the ICU for their cancer were consistently higher than 5-year survival rates reported for these cancer diagnoses in the Dutch population, that is, 13% for esophageal cancer, 57% for colorectal cancer, 4% for pancreatic cancer, 8% for biliary cancer, and 13% for lung cancer 37. Although these data cannot be compared directly, they support that ICU admission involves a selection of patients with relatively favorable therapeutic perspectives, for example because major surgery requiring postoperative ICU care will be limited to patients with early stage solid tumors carrying the better prognosis. The difference with long-term outcome with the general cancer population may even be higher considering that 5-year survival rate reported here relates to ICU admission date and not to date of cancer diagnosis. The finding that in our overall population cancer patients who were not admitted to the ICU did much better than patients who were admitted to the ICU (Fig.2A) can be explained by the fact that large patient groups with certain cancer diagnoses, such as breast and prostate cancer, have a relatively favorable prognosis and are rarely admitted to the ICU (Table2).

A limitation of our and previous studies that addressed long-term outcome after ICU admission 14,34–36 is that causes of death after hospital discharge were not specified and may be unrelated to ICU admission or cancer diagnosis. In addition, since 90% of all ICUs participate in the NICE registry we may have missed ICU referrals of some patients enrolled in our general population. The first day of the first DTC was used as starting time to determine time to ICU admission and death. In patients with more than one cancer diagnosis it would have been optimal to use the most relevant DTC for these calculations; however, it is not possible to retrieve this information from the DTC registry in a reliable manner. Additional limitations include the absence of information regarding severity scores in patients admitted to the ICU and cancer staging in the study population. Finally, it is uncertain whether the results, which were derived from four hospitals, are representative for the general population.

In conclusion, we report detailed information on the subgroup of cancer patients admitted to an ICU during a 5-year study period, comparing their demographics, diagnoses, and type of therapy with those in patients from the same population who did not receive ICU care. Our main findings were that only 6.4% of cancer patients were admitted to the ICU, that 26.9% of all ICU admissions of cancer patients were related to their cancer diagnosis, that most ICU admissions were after surgery and within one year of cancer diagnosis, and that in particular a diagnosis of gastrointestinal cancer was associated with ICU admission. The primary intent of our descriptive survey was not to change health care policies, but rather to provide epidemiologic data on cancer patients under treatment in general hospitals who during the course of their disease do or do not need ICU care. Patients with gastrointestinal malignancies, most notably esophageal cancer, were most often admitted to the ICU. In accordance, most patients entered the ICU for postoperative care. Considering the improved prognosis of cancer patients in general, combined with improved care on ICUs in general, surveys like presented here are temporal and likely to change at least in part depending on tumor type. Up-to-date knowledge of the outcome of cancer patients in relation to their need for ICU care can influence the discussion between and decision making by cancer specialists and intensivists.

## Conflict of Interest

None declared.

## Supporting Information

**Table S1.**Cumulative intensive care unit admission for the different age groups. **Table S2.** Cumulative intensive care unit admissions according to cancer diagnosis and type of treatment. **Table S3.** Logistic regression for ICU admission for different types of cancer.
