# Supplementary material for: Intensive care admission of cancer patients: a comparative analysis
Source: Cancer Med. 2015 Apr 18;4(7):966–76. doi: 10.1002/cam4.430 (PMC4529335; doi:10.1002/cam4.430)
Supplement: Supplementary file 1 [file cam40004-0966-sd1.docx]

**Table 1: Study Population Demographics**

|  | **All** | | **ICU admission** | | **no ICU admission** | | ***p^2^*** |
| --- | --- | --- | --- | --- | --- | --- | --- |
|  | **(% ICU admission)** | | **(% within this group)** | | **(% within this group)** | |  |
| All patients | 36,860 | (6.4) | 2,374 |  | 34,486 |  | *<0.001* |
| Total number of cancer diagnosis | 40,716 | (6.0) | 2,458 |  | 38,258 |  | *<0.001* |
| **Age (years)** |  |  |  |  |  |  |  |
| median  Interquartile range | 63  (52-72) | | 66  (57-73) | | 62  (52-72) | | *<0.001* |
| < 45 y | 5,186 | (2.8) | 143 | (6.0) | 5,043 | (14.6) | *<0.001* |
| 45-65 y | 11,063 | (5.8) | 646 | (27.2) | 10,417 | (30.2) | *<0.001* |
| >60-75 y | 14,323 | (8.1) | 1,158 | (48.8) | 13,165 | (38.2) | *<0.001* |
| >75 y | 6,288 | (6.8) | 427 | (18.0) | 5,861 | (17.0) | *0.214* |
| **Gender** |  |  |  |  |  |  |  |
| Male | 16,967 | (9.3) | 1,581 | (66.6) | 15,386 | (44.6) | *<0.001* |
| Female | 19,893 | (4.0) | 793 | (33.4) | 19,100 | (55.4) | *<0.001* |
| **Treatment (combinations^1^)** |  |  |  |  |  |  |  |
| Surgery | 7,661 | (11.8) | 904 | (36.8) | 6,757 | (17.7) | *<0.001* |
| Surgery and Chemotherapy | 2,044 | (9.3) | 190 | (7.7) | 1,854 | (4.8) | *<0.001* |
| Surgery and Radiation Therapy | 1,289 | (8.1) | 104 | (4.2) | 1,185 | (3.1) | *0.002* |
| Surgery, Chemotherapy, Radiation Therapy | 914 | (10.5) | 96 | (3.9) | 818 | (2.1) | *<0.001* |
| Chemotherapy | 3,426 | (6.2) | 213 | (8.7) | 3,213 | (8.4) | *0.137* |
| Chemotherapy and Radiation Therapy | 1,238 | (7.0) | 87 | (3.5) | 1,151 | (3.0) | *0.309* |
| Radiation Therapy | 7,502 | (2.3) | 169 | (6.9) | 7,333 | (19.2) | *<0.001* |
| Palliative care | 911 | (4.2) | 38 | (1.5) | 873 | (2.3) | *0.017* |
| Other/none | 15,731 | (4.2) | 657 | (26.7) | 15,074 | (39.4) | *<0.001* |

**^1^** Combinations of treatments are indicated independent of the order in which these were provided. ^2^ *p* ICU admission versus no ICU admission.

|  | **Number** | | | | **30 days ICU admission (%)^2^** | | | | **365 days ICU admission (%)^2^** | | | | **730 days ICU admission (%)^2^** | | | |
| --- | --- | --- | --- | --- | --- | --- | --- | --- | --- | --- | --- | --- | --- | --- | --- | --- |
|  |  | **(%)^1^** | **M & F** | | **All** | **M** | **F** | ***p*** | **All** | **M** | **F** | ***p*** | **All** | **M** | **F** | ***p*** |
| **Non-gender specific malignancy** |  |  |  |  |  |  |  |  |  |  |  |  |  |  |  |  |
| Lung cancer | 3,546 | (8.7) | 2,125 & | 1,421 | 1.6 | 2.0 | 1.1 | *0.474* | 5.3 | 6.2 | 4.0 | *0.061* | 6.3 | 7.5 | 4.4 | *0.003* |
| Head and neck cancer | 1,876 | (4.6) | 1,017 & | 859 | 0.9 | 0.9 | 0.9 | *0.676* | 3.7 | 4.7 | 2.6 | *0.131* | 5.2 | 6.6 | 3.5 | *0.001* |
| Colorectal cancer | 3,389 | (8.3) | 1,817 & | 1,572 | 2.3 | 2.3 | 2.3 | *0.660* | 8.4 | 9.9 | 6.7 | *0.263* | 10.4 | 11.9 | 8.7 | *0.003* |
| Pancreatic and biliary cancer | 2,759 | (6.8) | 1,418 & | 1,341 | 2.8 | 3.2 | 2.3 | *0.032* | 8.2 | 9.9 | 6.3 | *0.066* | 9.4 | 11.1 | 7.6 | *0.005* |
| Esophageal cancer | 1,661 | (4.1) | 1,274 & | 414 | 2.8 | 2.3 | 4.1 | *0.765* | 25.5 | 26.7 | 22.0 | *0.214* | 27.3 | 28.7 | 23.1 | *0.102* |
| Other types of GI-cancer | 1,323 | (3.2) | 803 & | 520 | 2.5 | 2.6 | 2.3 | *0.377* | 10.4 | 11.7 | 8.5 | *0.382* | 11.8 | 13.7 | 9.0 | *0.011* |
| Urinary tract cancer | 2,367 | (5.8) | 1,612 & | 755 | 0.9 | 1.0 | 0.8 | *0.452* | 4.6 | 5.1 | 3.4 | *0.547* | 5.5 | 6.2 | 4.1 | *0.011* |
| Melanoma | 491 | (1.2) | 225 & | 266 | 0.2 | 0.4 | 0.0 | *** | 0.8 | 1.8 | 0.0 | *0.050* | 1.5 | 2.5 | 0.7 | *0.059* |
| Sarcomas | 2,245 | (5.5) | 1,149 & | 1,096 | 0.4 | 0.6 | 0.2 | *0.170* | 1.9 | 2.3 | 1.4 | *0.017* | 2.8 | 3.6 | 1.9 | *0.019* |
| Hematological malignancy | 4,275 | (10.5) | 2,370 & | 1,905 | 1.6 | 1.9 | 1.3 | *0.675* | 4.7 | 5.8 | 3.3 | *0.332* | 6.2 | 7.7 | 4.4 | *<0.001* |
| Other types of cancer | 2,162 | (5.3) | 1,048 & | 1,114 | 1.4 | 1.8 | 1.1 | *0.293* | 3.3 | 4.0 | 2.6 | *0.193* | 4.2 | 5.4 | 3.0 | *0.201* |
|  |  |  |  |  |  |  |  |  |  |  |  |  |  |  |  |  |
| **Gender specific malignancy** |  |  |  |  |  |  |  |  |  |  |  |  |  |  |  |  |
| Breast cancer | 8,241 | (20.2) |  |  | 0.1 |  |  |  | 0.5 |  |  |  | 1.2 |  |  |  |
| Ovarian and endometrial cancer | 1,598 | (3.9) |  |  | 0.8 |  |  |  | 1.4 |  |  |  | 1.5 |  |  |  |
| Cervical cancer | 1,015 | (2.5) |  |  | 0.0 |  |  |  | 0.3 |  |  |  | 0.9 |  |  |  |
| Other types of gyn. cancer | 446 | (1.1) |  |  | 0.0 |  |  |  | 0.4 |  |  |  | 0.8 |  |  |  |
| Prostate cancer | 2,927 | (7.2) |  |  | 0.1 |  |  |  | 1.3 |  |  |  | 3.0 |  |  |  |
| Testicular cancer | 395 | (1.0) |  |  | 0.0 |  |  |  | 1.0 |  |  |  | 1.6 |  |  |  |

**Table 2: Cumulative Intensive Care Unit Admission stratified according to cancer diagnosis and gender**

^1^ % of all cancer diagnoses, ^2^ % of type of cancer in this cohort of 40,716 cancer diagnoses

*no comparison analysis is performed because the factor variable has only one value for every stratum. M is Male, F is Female.

**Table 3: Reason for admission in the ICU**

| **Type of ICU admission based on APACHE IV** | | |
| --- | --- | --- |
| **number of patients** | | **(%)** |
| Surgery for cancer | 579 | (23.6) |
| Other types of surgery |  |  |
| - Gastro-intestinal surgery | 322 | (13.1) |
| - Respiratory tract surgery (other than cancer) | 163 | (6.6) |
| - Cardiovascular surgery | 196 | (8.0) |
| - Other | 121 | (4.9) |
| Medical cancer | 80 | (3.3) |
| Other types of non-surgical admissions |  |  |
| - Cardiac | 173 | (7.0) |
| - Respiratory | 143 | (5.8) |
| - Infection / sepsis | 455 | (18.5) |
| - Thrombosis / hemorrhage | 91 | (3.7) |
| - Neurological | 44 | (1.8) |
| Other non-surgical causes for ICU admission | 87 | (3.5) |
| Missing | 4 | (0.2) |

**Supplementary Table 1: Cumulative Intensive Care Unit Admission for the Different Age Groups**

|  | **< 45 years** | | | **45-60 years** | | | **60-75 years** | | | **>75 years** | | |
| --- | --- | --- | --- | --- | --- | --- | --- | --- | --- | --- | --- | --- |
|  | **all** | **ICU (%)** | | **all** | **ICU (%)** | | **all** | **ICU (%)** | | **all** | **ICU (%)** | |
| **Non-gender specific malignancy** |  |  |  |  |  |  |  |  |  |  |  |  |
| Lung cancer | 114 | 7 | (6.1) | 990 | 70 | (7.1) | 1,649 | 119 | (7.2) | 793 | 26 | (3.3) |
| Head and neck cancer | 335 | 10 | (3.0) | 617 | 32 | (5.2) | 672 | 61 | (9.1) | 252 | 16 | (6.3) |
| Colorectal cancer | 169 | 4 | (2.4) | 809 | 61 | (7.5) | 1,467 | 155 | (10.6) | 944 | 134 | (14.2) |
| Pancreatic and biliary cancer | 190 | 5 | (2.6) | 833 | 66 | (7.9) | 1,248 | 139 | (11.1) | 488 | 42 | (8.6) |
| Esophageal cancer | 43 | 12 | (27.9) | 503 | 145 | (28.8) | 795 | 239 | (30.1) | 320 | 54 | (16.9) |
| Other types of GI-cancer | 136 | 8 | (5.9) | 394 | 46 | (11.7) | 568 | 68 | (12.0) | 225 | 35 | (15.6) |
| Urinary tract cancer | 219 | 7 | (3.2) | 580 | 31 | (5.3) | 954 | 74 | (7.8) | 614 | 38 | (6.2) |
| Melanoma | 131 | 2 | (1.5) | 176 | 3 | (1.7) | 132 | 5 | (3.8) | 62 | 10 | (16.1) |
| Sarcomas | 826 | 18 | (2.2) | 575 | 16 | (2.8) | 549 | 20 | (3.6) | 285 | 0 | (0.0) |
| Hematological malignancy | 990 | 45 | (4.5) | 1,261 | 111 | (8.8) | 1,336 | 117 | (8.8) | 688 | 32 | (4.7) |
| Other types of cancer | 723 | 14 | (1.9) | 465 | 20 | (4.3) | 580 | 42 | (7.2) | 394 | 7 | (1.8) |
| **Gender specific malignancy** |  |  |  |  |  |  |  |  |  |  |  |  |
| Breast cancer | 854 | 9 | (1.1) | 3402 | 40 | (1.2) | 3,032 | 50 | (1.6) | 953 | 23 | (2.4) |
| Ovarian and endometrial cancer | 162 | 0 | (0.0) | 528 | 9 | (1.7) | 661 | 12 | (1.8) | 247 | 12 | (4.9) |
| Cervical cancer | 464 | 1 | (0.2) | 341 | 2 | (0.6) | 137 | 5 | (3.6) | 73 | 0 | (0.0) |
| Other types of gynecological cancer | 51 | 2 | (3.9) | 108 | 0 | (0.0) | 129 | 2 | (1.6) | 158 | 0 | (0.0) |
| Prostate cancer | 7 | 0 | (0.0) | 485 | 10 | (2.1) | 1,881 | 85 | (4.5) | 554 | 23 | (4.2) |
| Testicular cancer | 283 | 4 | (1.4) | 91 | 1 | (1.1) | 14 | 2 | (14.3) | 7 | 0 | (0.0) |
| **Total** | **5,697** | **148** | **(2.6)** | **12,158** | **663** | **(5.5)** | **15,804** | **1,195** | **(7.6)** | **7,057** | **452** | **(6.4)** |

**Supplementary Table 2: Cumulative Intensive Care Unit admissions according to cancer diagnosis and type of treatment**

|  | **Surgery** | | | **Surgery and Chemotherapy** | | | **Surgery and Radiation Therapy** | | | **Surgery, Chemotherapy and Radiation Therapy** | | | **Chemotherapy and Radiation Therapy** | | |
| --- | --- | --- | --- | --- | --- | --- | --- | --- | --- | --- | --- | --- | --- | --- | --- |
|  |  |  |  |  |  |  |  |  |  |  |  |  |  |  |  |
|  | **all** | **ICU** | **(%)** | **all** | **ICU** | **(%)** | **all** | **ICU** | **(%)** | **all** | **ICU** | **(%)** | **all** | **ICU** | **(%)** |
| **Non-gender specific malignancy** |  |  |  |  |  |  |  |  |  |  |  |  |  |  |  |
| Lung cancer | 221 | 98 | (44.3) | 57 | 12 | (21.1) | 27 | 2 | (7.4) | 33 | 3 | (9.1) | 372 | 13 | (3.5) |
| Head and neck cancer | 246 | 27 | (11.0) | 32 | 0 | (0.0) | 112 | 24 | (21.4) | 40 | 7 | (17.5) | 52 | 4 | (7.7) |
| Colorectal cancer | 1,126 | 243 | (21.6) | 389 | 30 | (7.7) | 141 | 41 | (29.1) | 89 | 9 | (10.1) | 92 | 12 | (13.0) |
| Pancreatic and biliary cancer | 718 | 200 | (27.9) | 119 | 3 | (2.5) | 21 | 4 | (19.0) | 7 | 0 | (0.0) | 30 | 0 | (0.0) |
| Esophageal cancer | 347 | 247 | (71.2) | 179 | 102 | (57.0) | 44 | 31 | (70.5) | 132 | 79 | (59.8) | 112 | 13 | (11.6) |
| Other types of GI-cancer | 442 | 123 | (27.8) | 96 | 22 | (22.9) | 10 | 1 | (10.0) | 19 | 3 | (15.8) | 14 | 2 | (14.3) |
| Urinary tract cancer | 1,053 | 102 | (9.7) | 323 | 23 | (7.1) | 40 | 10 | (25.0) | 40 | 6 | (15.0) | 35 | 2 | (5.7) |
| Melanoma | 132 | 4 | (3.0) | 61 | 0 | (0.0) | 79 | 0 | (0.0) | 7 | 1 | (14.3) | 17 | 2 | (11.8) |
| Sarcomas | 463 | 18 | (3.9) | 25 | 13 | (52.0) | 89 | 10 | (11.2) | 29 | 2 | (6.9) | 30 | 0 | (0.0) |
| Hematological malignancy | 13 | 3 | (23.1) | 8 | 8 | (0.0) | 0 | 0 | (0.0) | 15 | 4 | (26.7) | 202 | 48 | (23.8) |
| Other types of cancer | 477 | 49 | (10.3) | 520 | 2 | (0.4) | 31 | 1 | (3.2) | 3 | 0 | (0.0) | 16 | 0 | (0.0) |
| **Gender specific malignancy** |  |  |  |  |  |  |  |  |  |  |  |  |  |  |  |
| Breast cancer | 587 | 11 | (1.9) | 526 | 8 | (1.5) | 317 | 5 | (1.6) | 472 | 6 | (1.3) | 224 | 5 | (2.2) |
| Ovarian and endometrial cancer | 744 | 18 | (2.4) | 142 | 6 | (4.2) | 76 | 1 | (1.3) | 8 | 0 | (0.0) | 9 | 0 | (0.0) |
| Cervical cancer | 354 | 1 | (0.3) | 11 | 0 | (0.0) | 143 | 1 | (0.7) | 33 | 0 | (0.0) | 13 | 0 | (0.0) |
| Other types of gynecological cancer | 201 | 2 | (1.0) | 0 | 0 | (0.0) | 37 | 0 | (0.0) | 0 | 0 | (0.0) | 0 | 0 | (0.0) |
| Prostate cancer | 758 | 40 | (5.3) | 20 | 2 | (10.0) | 216 | 9 | (4.2) | 7 | 1 | (14.3) | 23 | 1 | (43) |
| Testicular cancer | 64 | 3 | (4.7) | 30 | 1 | (3.3) | 12 | 0 | (0.0) | 5 | 0 | (0.0) | 13 | 1 | (7.7) |
| **Total** | **7,946** | **1,189** | **(15.0)** | **2,538** | **232** | **(9.1)** | **1395** | **140** | **(10.0)** | **939** | **121** | **(12.9)** | **1,254** | **103** | **(8.2)** |

**Continuing Supplementary Table 2: ICU admissions according to cancer diagnosis and type of treatment**

|  | **Chemotherapy** | | | **Radiation therapy** | | | **Palliative Care** | | | **Other, none** | | |
| --- | --- | --- | --- | --- | --- | --- | --- | --- | --- | --- | --- | --- |
|  |  |  |  |  |  |  |  |  |  |  |  |  |
|  | **all** | **ICU** | **(%)** | **all** | **ICU** | **(%)** | **all** | **ICU** | **(%)** | **all** | **ICU** | **(%)** |
| **Non-gender specific malignancy** |  |  |  |  |  |  |  |  |  |  |  |  |
| Lung cancer | 527 | 17 | (3.2) | 1,256 | 34 | (2.7) | 35 | 0 | (0.0) | 1,065 | 90 | (8.5) |
| Head and neck cancer | 64 | 4 | (6.3) | 547 | 13 | (2.4) | 12 | 0 | (0.0) | 800 | 69 | (8.6) |
| Colorectal cancer | 210 | 4 | (1.9) | 71 | 17 | (23.9) | 83 | 3 | (3.6) | 1,292 | 99 | (7.7) |
| Pancreatic and biliary cancer | 193 | 2 | (1.0) | 17 | 3 | (17.6) | 108 | 2 | (1.9) | 1,629 | 121 | (7.4) |
| Esophageal cancer | 147 | 19 | (12.9) | 82 | 5 | (6.1) | 45 | 3 | (6.7) | 712 | 90 | (12.6) |
| Other types of GI-cancer | 94 | 3 | (3.2) | 12 | 1 | (8.3) | 69 | 3 | (4.3) | 620 | 52 | (8.4) |
| Urinary tract cancer | 178 | 13 | (7.3) | 38 | 3 | (7.9) | 38 | 4 | (10.5) | 668 | 33 | (4.9) |
| Melanoma | 29 | 0 | (0.0) | 11 | 0 | (0.9) | 13 | 0 | (0.0) | 288 | 4 | (1.4) |
| Sarcomas | 97 | 7 | (7.2) | 513 | 10 | (1.8) | 11 | 0 | (0.0) | 949 | 15 | (1.6) |
| Hematological malignancy | 1,433 | 207 | (14.4) | 70 | 9 | (15.7) | 272 | 24 | (8.8) | 2,312 | 111 | (4.8) |
| Other types of cancer | 71 | 5 | (7.0) | 298 | 11 | (3.7) | 152 | 3 | (2.0) | 1,131 | 43 | (3.8) |
| **Gender specific malignancy** |  |  |  |  |  |  |  |  |  |  |  |  |
| Breast cancer | 292 | 3 | (1.0) | 3,955 | 65 | (1.6) | 36 | 0 | (0.0) | 1,840 | 27 | (1.5) |
| Ovarian and endometrial cancer | 28 | 4 | (14.3) | 85 | 4 | (4.7) | 11 | 0 | (0.0) | 501 | 6 | (1.2) |
| Cervical cancer | 7 | 0 | (0.0) | 84 | 1 | (1.2) | 7 | 1 | (14.3) | 363 | 4 | (1.1) |
| Other types of gynecological cancer | 58 | 0 | (0.0) | 10 | 0 | (0.0) | 0 | 0 | (0.0) | 199 | 3 | (1.5) |
| Prostate cancer | 74 | 1 | (1.4) | 494 | 28 | (5.7) | 24 | 1 | (4.2) | 1,340 | 58 | (4.3) |
| Testicular cancer | 75 | 2 | (2.7) | 5 | 0 | (0.0) | 1 | 0 | (0.0) | 191 | 1 | (0.5) |
| **Total** | **3,577** | **291** | **(8.1)** | **7,548** | **204** | **(2.7)** | **917** | **44** | **(4.8)** | **15,900** | **826** | **(5.2)** |

**Supplementary Table 3: Logistic regression for ICU admission for different types of cancer**

|  | **Odds ratio^1^**  **(95% Confidence Interval)** | ***p*** |
| --- | --- | --- |
| **Non-gender specific malignancy** |  |  |
| Lung cancer | Reference |  |
| Head and neck cancer | 0.96 (0.77 – 1.19) | *0.707* |
| Colorectal cancer | 0.94 (0.79 – 1.12) | *0.468* |
| Pancreatic and biliary cancer | 1.06 (0.88 – 1.28) | *0.527* |
| Esophageal cancer | 3.27 (2.74 – 3.89) | *<0.001* |
| Other types of GI-cancer | 1.18 (0.96 – 1.45) | *0.126* |
| Urinary tract cancer | 0.48 (0.39 – 0.60) | *<0.001* |
| Melanoma | 0.23 (0.12 – 0.43) | *<0.001* |
| Sarcomas | 0.39 (0.29 – 0.51) | *<0.001* |
| Hematological malignancy | 0.62 (0.08 – 5.15) | *0.661* |
| Other types of cancer | 0.75 (0.59 – 0.95) | *0.018* |
| **Gender specific malignancy** |  |  |
| Breast cancer | 0.20 (0.15 – 0.25) | *<0.001* |
| Ovarian and endometrial cancer | 0.21 (0.15 – 0.31) | *<0.001* |
| Cervical cancer | 0.09 (0.04 – 0.18) | *<0.001* |
| Other types of gynecological cancer | 0.10 (0.04 – 0.26) | *<0.001* |
| Prostate cancer | 0.26 (0.21 – 0.32) | *<0.001* |
| Testicular cancer | 0.19 (0.09 – 0.39) | *<0.001* |

^1^ The calculated odds ratios were adjusted for gender, therapy combinations, type of cancer and age by adding these covariates in the logistic regression model with ICU admission as the dependent variable.
